# Supplementary material for: Positive Effects of Argon Inhalation After Traumatic Brain Injury in Rats
Source: Int J Mol Sci. 2024 Nov 26;25(23):12673. doi: 10.3390/ijms252312673 (PMC11640893; doi:10.3390/ijms252312673)
Supplement: Supplementary file 1 [file ijms-25-12673-s001.zip › ijms-3310737-supplementary.pdf]

Supplementary for figure 6:

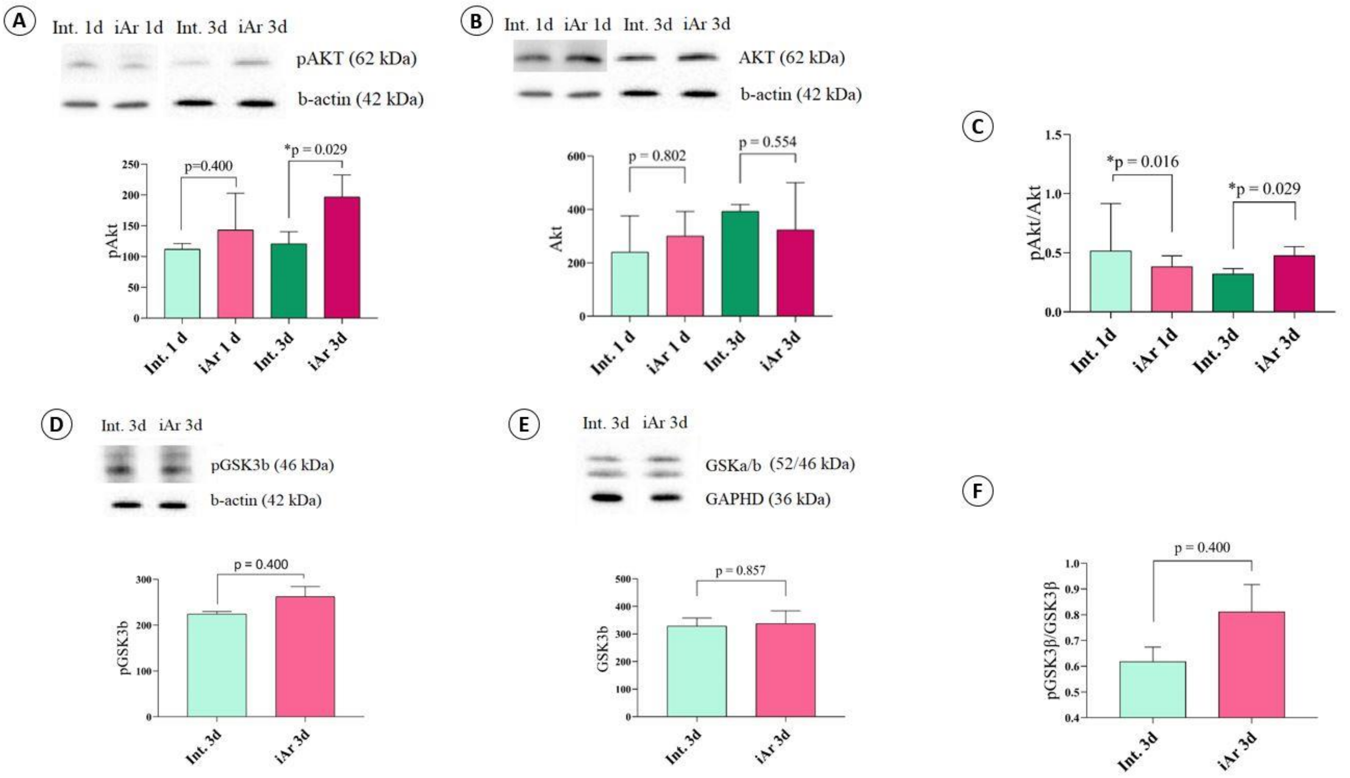

Figure S1. Influence of argon on preconditioning: Representative immunoblots and densitometry results of phosphorylated forms of proteins AKT (pAKT) (**A**), total AKT (**B**) and protein forum ratio pAKT/AKT (**C**); phosphorylated forms of proteins GSK3β (pGSK3β) (**D**), total GSK (**E**)and protein forum ratio pGSK/GSK (**F**) after argon exposure. Signals were normalized to β-actin or GAPDH as the loading control. The observed molecular weights of the proteins are labeled. The data are presented as median (inter quartile range). * - $p < 0.05$ vs. the TBI group based on the Mann–Whitney U-test.
